# Exploring Soil Factors Determining Composition and Structure of the Bacterial Communities in Saline-Alkali Soils of Songnen Plain

**DOI:** 10.3389/fmicb.2019.02902

**Published:** 2020-01-14

**Authors:** Shuang Wang, Lei Sun, Ning Ling, Chen Zhu, Fengqin Chi, Weiqun Li, Xiaoyu Hao, Wu Zhang, Jingyang Bian, Lei Chen, Dan Wei

**Affiliations:** ^1^Key Lab of Soil Environment and Plant Nutrition of Heilongjiang Province, Heilongjiang Fertilizer Engineering Research Center, Institute of Soil Fertilizer and Environment Resources, Heilongjiang Academy of Agricultural Sciences, Harbin, China; ^2^Jiangsu Provincial Key Lab for Organic Solid Waste Utilization, Nanjing Agricultural University, Nanjing, China; ^3^Heihe Branch of Heilongjiang Academy of Agricultural Sciences, Heihe, China; ^4^Daqing Branch of Heilongjiang Academy of Agricultural Sciences, Daqing, China; ^5^Institute of Plant Nutrition and Resources, Beijing Academy of Agriculture and Forestry Sciences, Beijing, China

**Keywords:** bacterial community, driving force, electrical conductivity, saline-alkali soil, Songnen Plain

## Abstract

Songnen Plain is originally one of the three major glasslands in China and has now become one of the three most concentrated distribution areas of sodic-saline soil worldwide. The soil is continuously degraded by natural and anthropogenic processes, which has a negative impact on agricultural production. The investigation of microbial diversity in this degraded ecosystem is fundamental for comprehending biological and ecological processes and harnessing the potential of microbial resources. The Illumina MiSeq sequencing method was practiced to investigate the bacterial diversity and composition in saline-alkali soil. The results from this study show that the change in pH under alkaline conditions was not the major contributor in shaping bacterial community in Songnen Plain. The electrical conductivity (EC) content of soil was the most important driving force for bacterial composition (20.83%), and the second most influencing factor was Na^+^ content (14.17%). Bacterial communities were clearly separated in accordance with the EC. The dominant bacterial groups were *Planctomycetes*, *Proteobacteria*, and *Bacteroidetes* among the different salinity soil. As the salt concentration increased, the indicators changed from *Planctomycetes* and *Bacteroidetes* to *Proteobacteria* and *Firmicutes*. Our results suggest that *Proteobacteria* and *Firmicutes* were the main indicator species reflecting changes of the main microbial groups and the EC as a key factor drives the composition of the bacterial community under alkaline conditions in saline-alkali soil of Songnen Plain.

## Introduction

Salinity and/or sodicity is one of the main problems causing soil degradation, which is a grievous environmental problem with negative impacts on agricultural sustainable development. All over the world, over 800 million ha of land is estimated to be affected by salinity, which includes saline and alkaline soil (Yadav et al., 2011). It is reported that ~20% of the agricultural land worldwide is salt affected, and there is an increase in saline land of 1–1.5 × 10^6^ ha worldwide every year (Sumner, 2000; Munns and Tester, 2008). If this continues, about half of cultivable land will be taken out of production by the middle of the 21st century (Mahajan and Tuteja, 2005). As one of the three most concentrated alkaline soil distribution areas worldwide, the Songnen Plain is located in the northeast of China (Wang et al., 2009). In this region, several environmental problems such as soil salinization, alkalinization, and desertification occur due to soil parent material, hydrological conditions, and overgrazing (Zhou et al., 2011). The annual rainfall exceeds the potential evaporation and poor management such as overgrazing and irrational utilization are common in the area (Gao and Liu, 2010; Liu et al., 2011a); thus, the affected alkali-saline area has been increasing in size by 20,000 ha per year (Zhang et al., 2015). Not only about two-thirds of the land in this area is salinized, but also increased by 1.5–2% annually (Ma et al., 2015). Under increase aridity and soil alkalization, large areas of croplands have been abandoned (Shang et al., 2003) and large proportions of grasslands have degraded seriously to unprecedented levels (Li et al., 2014).

Soil microorganisms participate in multiple aspects in the adjustment of ecological processes, for instance, degradation of organic matter, nutrient element transformations, enzyme production, and maintenance of soil quality (Sleator et al., 2008). Given that microorganisms are rapidly affected by the changes of their environment (Jiang et al., 2012), some biological characteristics (such as microbial diversity, composition, and structure) of soil are often considered to be sensitive and early indicators of dynamic environmental changes and soil ecological stress status (Li et al., 2011; Liu and Kang, 2014). For instance, soil microbial diversity was shaped by land-use changes, such as urbanization, agriculture, deforestation, and desertification. Many studies have shown that current environmental factors, for example, nutritional status, metalloid contamination, soil pH, the plant secretion (Hansel et al., 2008; Rousk et al., 2010; Xiong et al., 2010), and geographic distance (Xiong et al., 2012), influence structure and composition of microbial community.

Among the environmental factors, the pH was proposed to be a driver force for bacterial horizontal distribution in the soil (Shen et al., 2013; Liu et al., 2014). As soil salinization and alkalinization frequently co-occur, meta-analysis has been conducted merely on microbial diversity and composition (including bacteria and archaea) in saline soil habitats (Lozupone and Knight, 2007; Ma and Gong, 2013). Salinity is a dominant factor in determining bacterial community composition (Lozupone and Knight, 2007), however, relatively little is known about how the bacterial community composition response to the salinity gradients. Especially, the microbial structure under different salinities at similar pH has not been investigated.

Several recent studies on the composition of soil microbial diversity in saline soils revealed that soil salinization had negative effects not merely on soil biochemical properties, but also on the structure of microbial communities (Foti et al., 2007; Yuan et al., 2007; Hidri et al., 2013; Wang et al., 2014; Zhang et al., 2015). Regarding the soil microbial community in saline soils of Songnen Plain, only some representative new species of halophilic and halotolerant bacteria and archaea have been reported by pure culture methods (Wu et al., 2008; Wang et al., 2010; Liu et al., 2011b; He et al., 2014; Pan et al., 2016). These microorganisms have gradually formed adaptations, including unique structures and physiological functions, such as the accumulation of osmotic adjustment-related substances (Yan and Marschner, 2012). However, physical and chemical properties in saline-alkaline environments and microbial composition in Songnen Plain have not been sufficiently explored.

Systematic analysis of microbial diversity and composition in saline-alkaline soil of Songnen Plain is essential for gaining insight into the biological and ecological processes, saline adaption mechanisms, and digging the potential microbial resources from such environments. We collected 29 soil samples with different salinities across Songnen Plain and performed high throughput sequencing (Illumina MiSeq sequencing) to investigate the microbial diversity and composition in this under-studied system and to identify the key factors controlling the distribution of bacterial communities. The aim was to clarify the direct effects of environmental factors in shaping bacterial communities under geographic scale.

## Materials and Methods

### Site Description

The study area locates in the Songnen Plain (42°30′–51°20′N, 121°40′–128°30′E), Northeast China and belongs to a transitional zone between semi-humid and semi-arid regions, and is typically influenced by continental monsoon climate with mean annual temperature of 4.7°C (Shang et al., 2003; Yang et al., 2010). The average annual evaporation in this region is four times greater than the annual precipitation. The groundwater average mineral content is 2–5 g/L, with a maximum of 10 g/L, and the major anions present are CO_3_^2−^ and HCO_3_^−^ (Zhang, 2014). The saline-sodic soils are characterized by a high pH (up to 10) and a large exchangeable Na percentage (Chi and Wang, 2010). The dominant zonal soils include meadow carbonate chernozem, deep chernozem, sodium carbonate-salinized soil, and dark chestnut soil. These soils are mainly distributed in the west part of Jilin and Heilongjiang provinces (Chi et al., 2011), including Zhenglai, Da’an, Changling, Qianguo, and Tongyu prefectures in Jilin province, and Zhaoyuan, Zhaozhou, Dumeng, Daqing, and Anda prefectures in Heilongjiang province (Wang et al., 2009). There are no naturally growing tree species because of salinization, only some salt/sodium-tolerant grass species such as *Leymus chinensis*, *Puccinellia tenuiflora*, and *Suaeda corniculata* are able to grow in the study area.

### Site Selection and Soil Sampling

**Figure 1:**
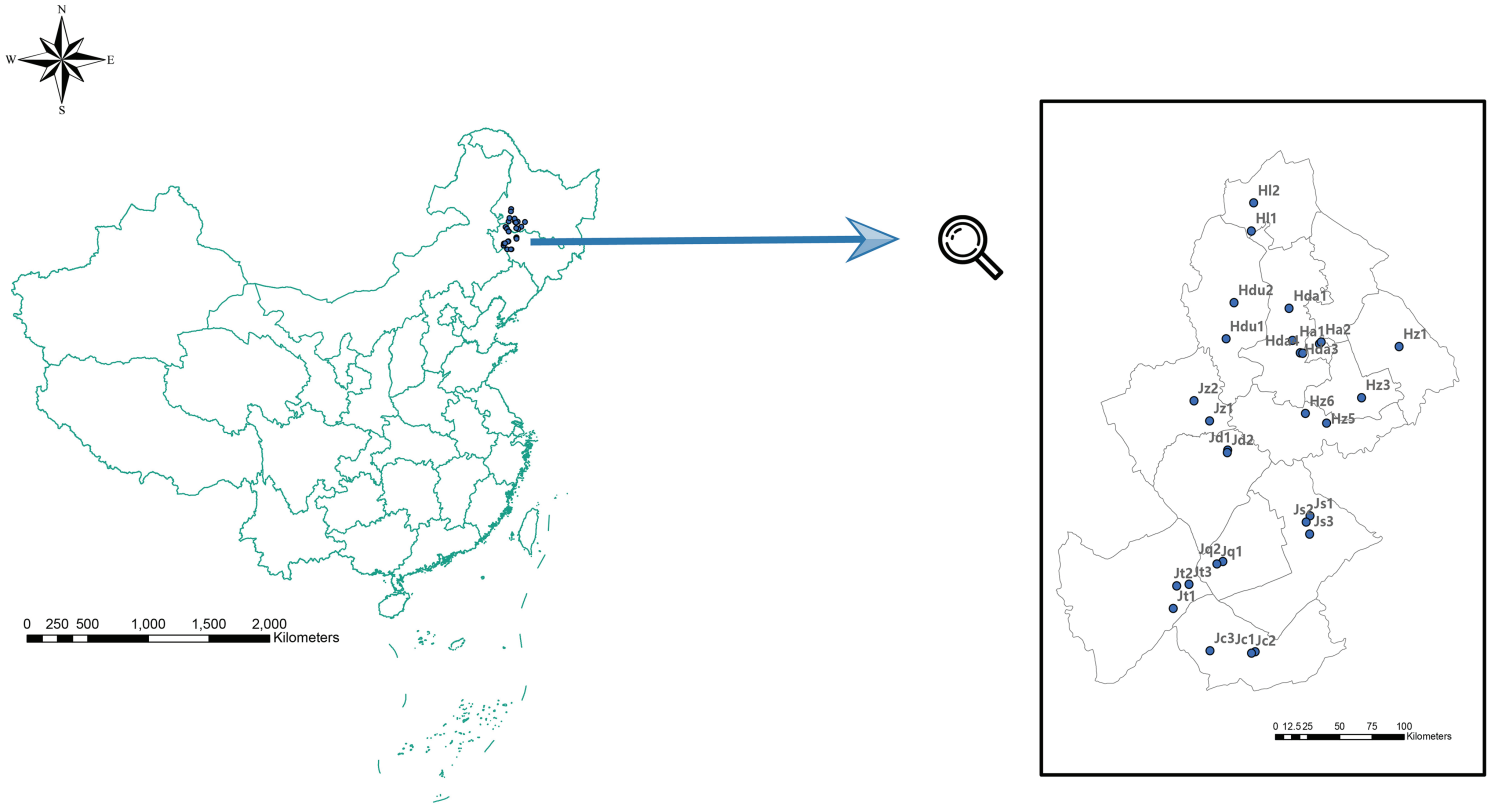
Sampling positions in Jilin Province [including Zhenglai (Jz), Da’an (Jd), Qianguo (Jq), Changling (Jc), Songyuan (Js), and Tongyu (Jt) prefectures], and in Heilongjiang Province [including Dumeng (Hdu), Lindian (Hl), Daqing (Hda), Anda (Ha) Zhaodong (Hz1), and Zhaozhou (Hz3) prefectures].

A total of 29 soil samples (with a mean depth of 0–15 cm) with GPS located site information from 12 counties (cities) across Jilin and Heilongjiang provinces were randomly collected in September 2013 (Figure 1). At each site, the analyzed soil samples mixture was from three soil samples collected in the vertices of 1 m side equilateral triangle. Then, the soil sample mixture from each site was divided into two subsamples: one was air dried, 2 mm sieved for soil physical and chemical analysis, and the other subsample was stored at −80°C for subsequent high-throughput sequencing.

### Soil Physical and Chemical Analysis

Soil organic carbon (SOC) was measured on a Total Organic Carbon (TOC) Analyzer (Multi N/C2100, Analytik-Jena, Germany). The electrical conductivity of the saturated paste extraction (EC) was measured using a conductivity meter (DDS-307A, REX, Shanghai), and pH was determined with air-dried soil (soil:water, 1:5) by pH meter (PHS-3C, REX, Shanghai). Soil exchangeable sodium(ammonium acetate exchange method) was determined by an atomic absorption spectrophotometer (TAS-990, Persee, Shanghai). Potentiometric titration was used to determine CO_3_^2−^, HCO_3_^−^, Cl^−^, and SO_4_^2−^ with air-dried soil (soil:water, 1:5). The cations Ca^2+^ and Mg^2+^ were detected using Ethylenediaminetetraacetic acid (EDTA) titration and Na^+^ and K^+^ were measured using an atomic absorption spectrophotometer (TAS-990, Persee, Shanghai). Total content of dissolved salt (TDS) was determined by the drying-weighing method. The soil:water 1:5 tilt rate was placed in an oven at 105°C until constant weight was reached.

### Soil DNA Extraction

The total DNA was extracted from 0.25 g soil samples using the Power Soil DNA Isolation Kit (Mo Bio Laboratories Inc., Carlsbad, CA, USA), followed by electrophoresis using a 1% agarose gel. The quality and quantity of DNA extracts (final volume, 100 μl) were examined using a Nano Drop spectrophotometer (Nano Drop Technologies, Wilmington, DE, USA).

### Illumina Sequencing for Communities of Bacteria

The primer pair 515F (5′-GTGCCAGCMGCCGCGG-3′)/907R (5′-CCGTCAATTCMTTTRAGTTT-3′) was used to amplify the V4-V5 region of bacterial 16S rRNA genes (Yao et al., 2017). A 6-bp unique barcode unique to each sample was added into the reverse primers. Amplicon sequencing was performed using the Illumina MiSeq platform at Majorbio Inc. (Shanghai, China).

The sequencing data analysis was performed according to previous study (Zhu et al., 2016). Briefly, raw high-throughput sequencing data was processed using QIIME toolkit and the UPARSE pipeline (Caporaso et al., 2010; Edgar, 2013). After filtering DNA sequences using quality files, the remaining sequences were trimmed to remove barcodes and forward primers. The low quality (quality score < 20, length < 300 bp) sequences were excluded. The sequencing data were pre-treated to remove the chimeras from the datasets. After optimizing the sequences, the UPARSE pipeline was used to make an operational taxonomic unit (OTU) table. The identity threshold to bin the sequences into OTUs was 97%, and the most abundant sequence from each OTU was selected as the representative sequence for that OTU. The assignment of taxonomic data to microbial representative sequences was based on the Ribosomal Data Project (RDP) database. All sequencing data were deposited in the NCBI Sequence Read Archive (SRA) database (accession number: SRP172399).

### Statistical Analysis

Analysis of variance was performed for each measured soil variable, and variance was compared between groups by a Fisher’s least significant difference test (*α* = 0.05). The richness index (Chao1 index), α-diversity (Shannon diversity) index, and rarefaction curves of soil samples were calculated by QIIME with normalized data. Non-metric multidimensional scaling (NMDS), and one-way PERMANOVA were used to analyze the β-diversity of bacteria in different treatments according to Bray-Curtis distance and Canonicla Correlation Analysis (CCA) was used to examined the relationships between the environmental factors and bacterial community structure with the R (2.15.3) packages ape and vegan. Aggregated boosted tree (ABT) analysis (De'ath, 2007), was performed using the gbmplus package (with 500 trees used for the boosting, 0.02-fold shrinkage rate and three-way interactions) to determine the relative influence of environmental variables on bacterial community composition (NMDS axis 1).

Network analyses were used to dissect the interrelationship and interaction between bacterial species along the different electrical conductivity (EC) gradients. All pair wise Pearson correlation coefficients were calculated by Mothur (version 1.29.2) for analysis of the networks. The correlation coefficient of Pearson correlation was further filtered with the cut-off as an absolute value of 0.6–0.93. After applying multiple hypothesis correction by the BH method (Benjamini and Hochberg, 1995), edges with adjusted values of *p* below 0.05 were kept and were further used to improve the veracity of the networks. Interactive networks were visualized by Gephi with a Fruchterman-Reingold layout (Bastian et al., 2009). The average clustering coefficient, average path length, and modularity of the network were calculated (Newman, 2006). We referred to the active species as the species that most strongly interacts with the other species within the networks (Magurran and Henderson, 2003; Montoya et al., 2006; Barberán et al., 2012). According to the network analysis, we selected the first 10 hubs under different salinity treatments. Times of iteration were 10,000. The indicator status of OTUs from each of the salinity treatments was assessed, and indicator of a treatment was conducted under the significance threshold of 0.05.

## Results

### Physical and Chemical Properties for All Collected Samples

To explore the soil factors that affect bacterial community composition, we first survey the physical and chemical properties in over 29 soil samples in different salinities collected from Jilin and Heilongjiang provinces (Figure 1). In all the studied sites, soil samples showed differences in pH. One sample from Ha2 had a pH of 8.84, whereas the pH of others was ranged from 9.89 to 10.66. However, the remaining physical and chemical properties, including various ion concentrations (Cl^−^, Na^+^, SO_4_^2−^, CO_3_^2−^, HCO_3_^−^, Ca^2+^, Mg^2+^, and Na^+^), SOC, EC, and SAR, varied more substantially than the pH. In particular, the lowest concentration of SO_4_^2−^ was 0.480 g/kg from Ha1, and the highest was 21.6 g/kg in Hda3, a difference reached 48 times. The same conspicuous change was observed in EC, the highest EC (Hda4: 10.867 ms/cm) was 26.3 times higher than the lowest value (Jc1: 0.413 ms/cm) (Supplementary Table S1).

### Bacterial Community Analysis

**Figure 2:**
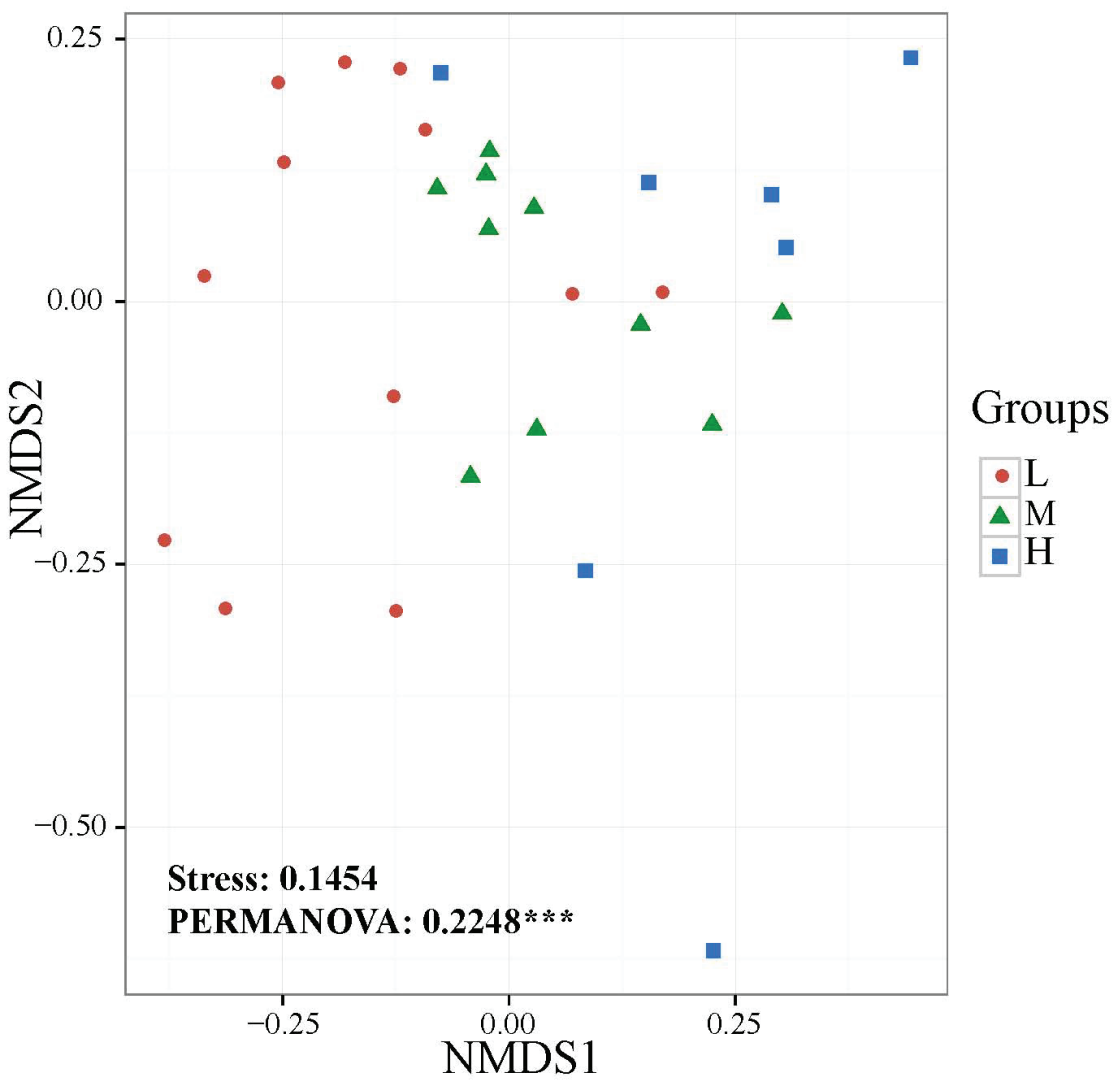
Non-metric multidimensional scaling (NMDS) ordination plot of soil bacteria community structure based on the number of OTUs detected by sequencing.

**Figure 3:**
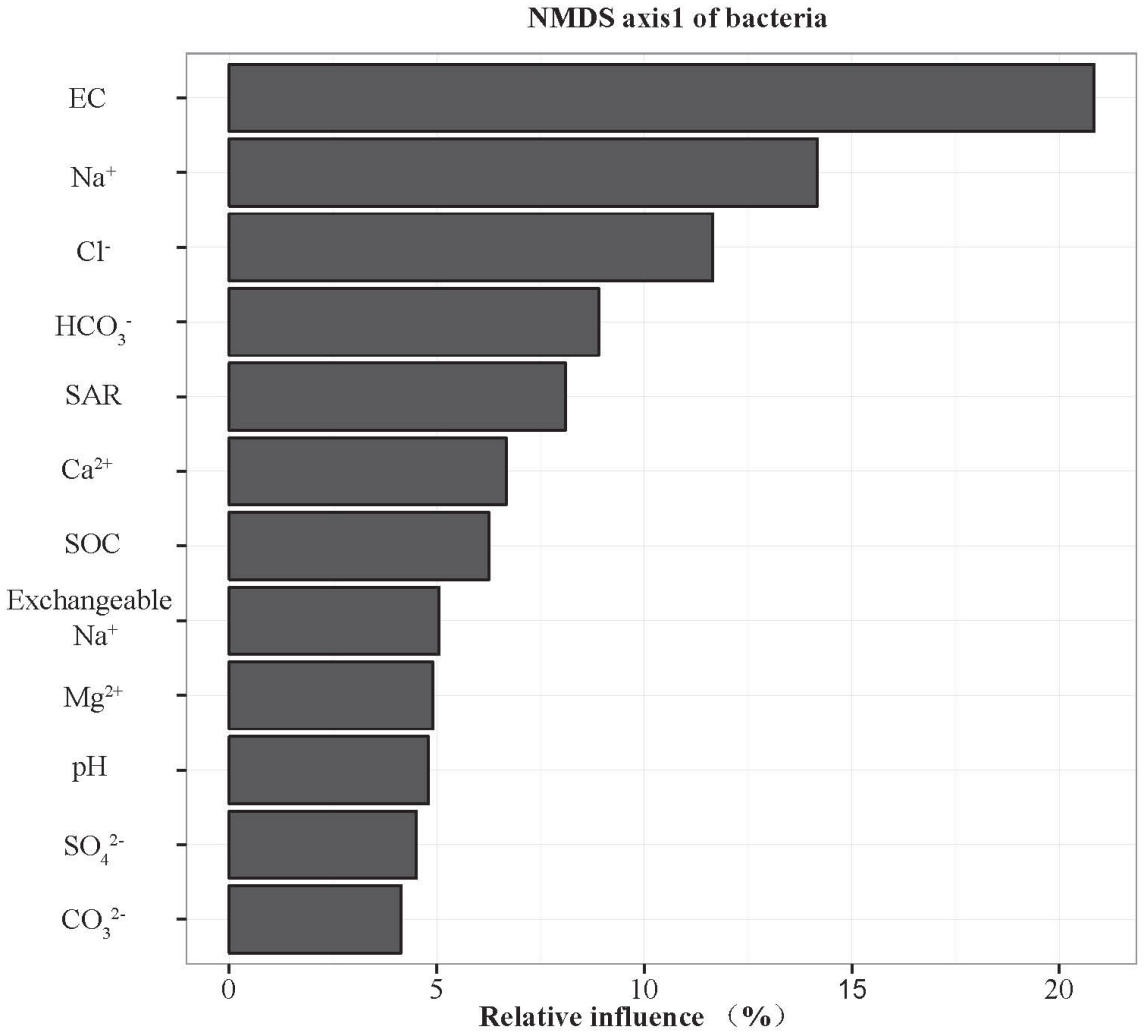
Relative variable importance plot (%) of environmental driving factors for composition of bacteria by ABT models.

**Figure 4:**
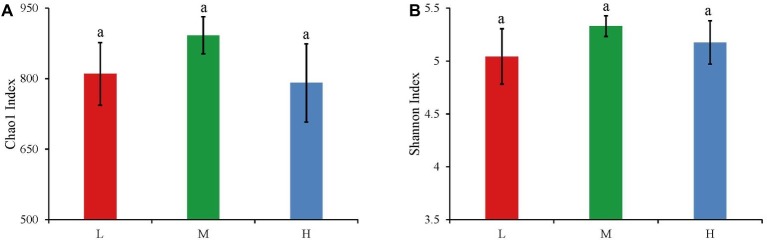
The Chao1 index **(A)** and Shannon index **(B)** of bacteria in three salinity rates. The values are the means of three replicates, error bars represent standard errors (L: *n* = 12; M: *n* = 10; H: *n* = 7), and different letters above the bars indicate significance differences at the *p* < 0.05 level. The L, M, and H treatments represent EC of 0–2, 2–4, and >4 ms/cm, respectively.

The widespread change of the physical and chemical properties leads us to ask whether these properties would be associated with bacterial community in the salinity soils. The soil bacterial communities under different salinization levels were compared by sequencing of the bacterial 16S rRNA amplifications. Therefore, we sought to identify environmental factors that contribute to the ecological variation of bacterial community by analyzing characteristics of different soil samples (Supplementary Table S1), and creating ABT models to evaluate the relative impact of environmental factors on the bacterial composition NMDS axis 1. The NMDS result showed that the bacterial community structure was significantly separated in three EC levels (PERMANOVA, *F* = 0.2248, *p* < 0.001) (Figure 2). Soil EC was the most important driving force for microbial composition (20.83%, Figure 3), and the second most influencing factor was Na^+^ (14.17%) followed by Cl^−^. Given the key role of salinity in shaping the bacterial community, we tentatively divided all samples into three groups depending on the EC level as low (L)-, medium (M)- and high (H)-level treatment. The L, M, and H treatments represent EC of 0–2, 2–4, and >4 ms/cm, respectively. However, there was no significant diffidence in the α-diversity, including Chao1 index and Shannon index, among the three different salinity levels (Figure 4).

### The Co-occurrence of Bacteria in Three-Salinity Gradient Treatments

**Figure 5:**
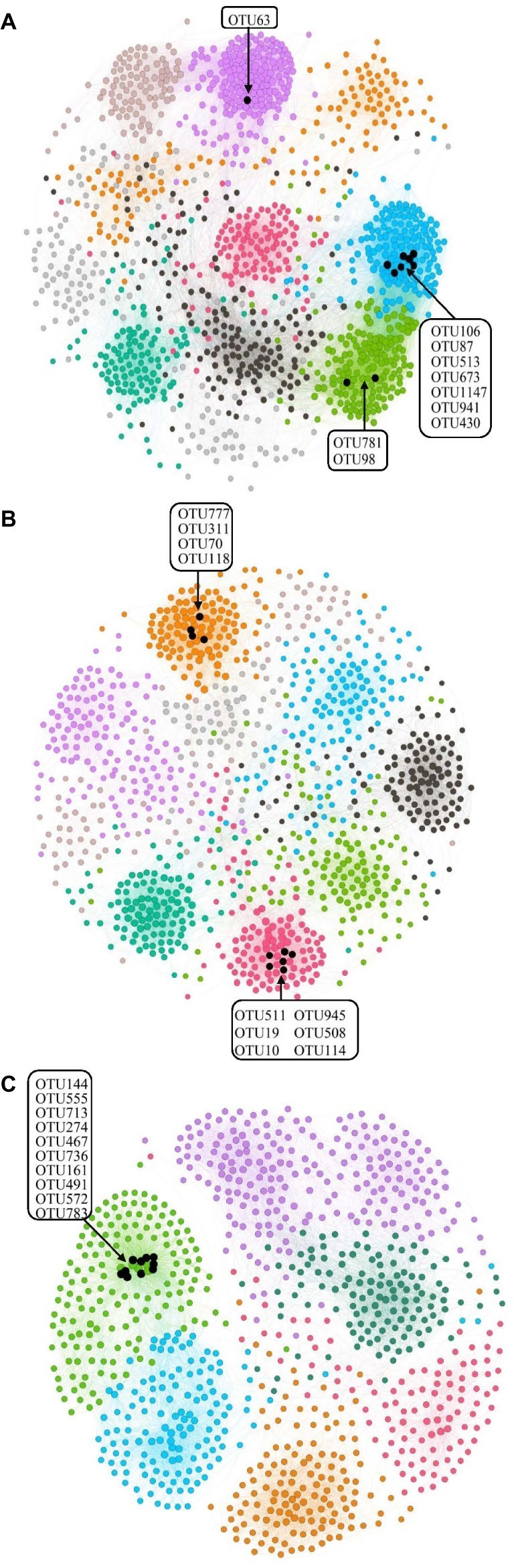
Network analysis of the three different salinity rates treatments based on Pearson correlations. The different color edges belong to different modules. **(A)** Low-salinity treatment (EC: 0–2) with modularity resolution of 0.72 and 10 modules; **(B)** middle-salinity treatment (EC: 2–4) with modularity resolution of 0.82 and 9 modules; and **(C)** high-salinity treatment (EC: >4) with modularity resolution of 0.74 and 7 modules. The black nodes indicate the OTUs belonging to active hubs in the network.

Bacterial species usually work corporately to regulate soil properties. Based on the abovementioned statement about dividing the samples into three groups, we divided all samples into three salinity treatments (L, M, and H treatments) and the three treatment groups were used for network analysis. The network parameters of the three salinity levels were showed significant differences (Figure 5). In the L treatment, the network had 1,208 nodes and 29,183 edges, and the modularity was 0.72, with 10 modules. For the M treatment, the network presented 982 nodes and 12,819 edges, and the modularity was 0.82, with 9 modules. Finally, 878 nodes and 10,871 edges were found in the H treatment, for which the modularity was 0.74, with 7 modules. Nodes, edges, modularity, and modules all declined with increasing salinity, suggesting that a higher concentration of salt led to reducing connectivity of the bacterial network (Figure 5).

**Figure 6:**
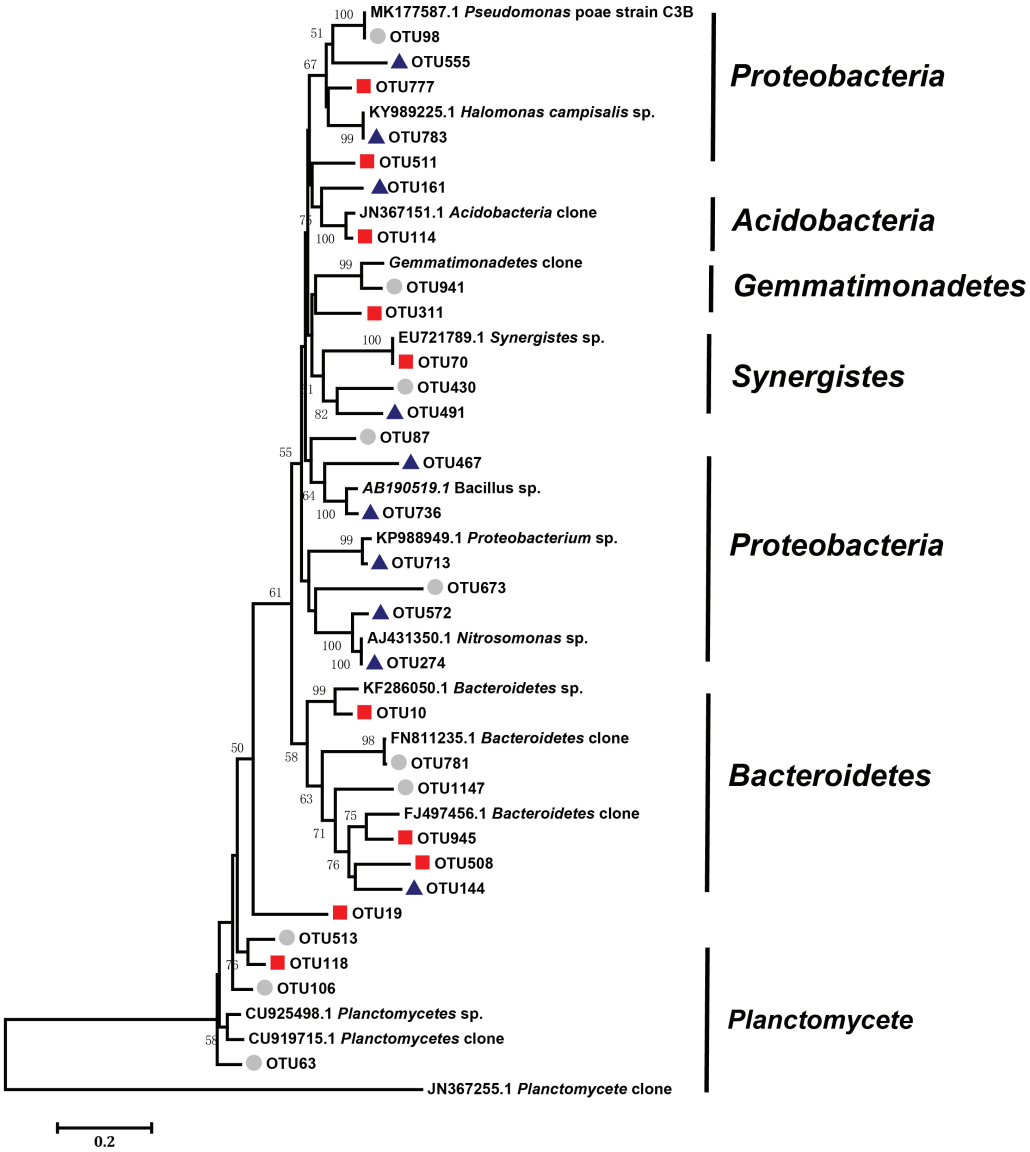
Neighbor-joining analysis of key OTUs forms the networks of three different salinity treatments (low, middle, and high). Bootstrap values higher than 40% are indicated at branch nodes. The scale bar represents 2% nucleic acid sequence divergence. The key OTUs as circles represent OTUs in the low-salinity treatment (EC: 0–2); the key OTUs as squares represent OTUs in the middle-salinity treatment (EC: 2–4); and the key OTUs as triangles represent OTUs in the high-salinity treatment (EC:>4).

We defined the 10 nodes with largest number of edges as network hubs, which were active mediators in the bacterial community network (Supplementary Table S2). Taken as a whole, *Planctomycetes*, *Proteobacteria*, and *Bacteroidetes* were the three dominant phyla among the different salinity rate treatments, including 63.3% network hubs at the phylum level (Figure 6). Half of the network hubs in the L treatment belonged to *Planctomycetes* and *Bacteroidetes*, such as OTU513, OTU106, OTU63, OTU781, and OTU1147. The hubs of M treatment had similar phylogenetic classifications to the L treatment, with half assigned to *Planctomycetes* and *Bacteroidetes*. However, at the highest salinity, more than half hubs were classified as *Proteobacteria*, for instance, OTU713, OTU274, OTU572, OTU555, and OTU783. However, only one indicator (OTU144) belonged to the classification of phyla at low salt concentrations, while a second indicator (OTU777) belonged to the classification of phyla in moderate salt concentrations, which were identified as *Planctomycetes* and *Bacteroidetes*, respectively. As the salt concentration increased, the indicators changed from *Planctomycetes* and *Bacteroidetes* to *Proteobacteria* and *Firmicutes*. Supplementary Figure S2 shows that the *Rhodospirillaceae*, belonging to a family of *Proteobacteria*, had a strong correlation with the EC (*R*^2^ = 0.3365, *p* < 0.001). At the higher taxonomic level, the genus of *Marinicella*, assigned to *Proteobacteria*, also had a positive correlation with EC. The results revealed that multiple microbes belonging to *Proteobacteria* are well adapted to the high-salinity environment.

## Discussion

### Driving Force for Microbial Community Composition in Saline Soils

We collected a total of 29 soil samples from Jilin and Heilongjiang provinces to investigate the distribution characteristics of microbes and reveal the general rule of microbial community composition under different salinities. Saline soils are characterized by high salt concentrations as well as an uneven temporal and spatial water distribution. The high salt concentrations shape the special environment patterns for microbes, causing the microbes in saline soils to vary from those found in the non-saline environment (Canfora et al., 2014). Firstly, we have analyzed the physical and chemical properties among the samples with different salinities. Although pH is a potential important determinant of salinity, we found that pH does not vary substantially among these samples, while the concentration of CO_3_^2−^ varies greatly, from 0.01 to 2.82 g/kg. Besides, the concentration of other ions, SOC, EC, and SAR are all different to some extent in the 29 soil samples, encouraging us to investigate whether the variation of physical and chemical properties was associated with salinity.

Identifying environmental factors contributing to the variation of microbial communities is a central aim in ecology. Several studies have shown that environmental factors, including soil pH (Rousk et al., 2010) and trophic status (Hansel et al., 2008; Xiong et al., 2010) affect the microbial community. As pH is the most important driver in bacterial community change (Shen et al., 2013; Liu et al., 2014), the explanation of pH for community variation only accounted for 4.80% in this study. ABT models were used to analyze the influence of the environmental factors on the bacterial community composition. EC content of soil was the most important driving force for microbial community composition (Figure 3) rather than soil pH. In CCA analysis, the EC value was the highest factor explaining the bacterial community by Monte Carlo permutation (Supplementary Table S3). This result showed gradient distribution of salinity along our sampling sites. Thus, EC could be the major factor controlling the differences. It has been reported recently that both low and high EC will affect bacterial growth in high-salinity soils (Rath et al., 2019), the influence of EC might be different among bacterial species, supporting our finding that EC is a causal factor for bacterial community in our salinity soil samples.

A previous study has shown that bacterial composition was associated with pH changes in both acidic soils and alkaline lake sediment (Xiong et al., 2012). While the current study shows that pH is unlikely to be the principal contributor to the considerable microbial community changes according to the ABT model. It should be noted that effect of pH in shaping bacterial communities is affected by local features. It is well known that salinization consists of salt accumulation by one or more natural processes including high salt content of the parent material or in groundwater, and human interventions such as inappropriate irrigation practices or inappropriate use of fertilization regimes. However, no matter which way, Na^+^ is a type of salt, which is the main component. For alkaliphilic bacterial taxa, the Na^+^ could serve as important proton replacement to cope with the high external pH (Canfora et al., 2014). As the relative abundance of these microbes are associated with the carbon mineralization rate in the soil (Fierer et al., 2007), we speculate the changes of dominant taxa that occur across the salinity gradient probably play a role in regulating ecosystem functions.

### Changes in Diversity and Structure of Microbial Community in Saline Soils

In general, soil microbial diversity changes distinctly in response to environmental variations (Liu and Kang, 2014). However, in this study, the α-diversity did not change remarkably, whereas there was significant variation in β-diversity under the different salinity. Moreover, some studies have reported that α-diversity and β-diversity did not change simultaneously β-diversity transforms prior to α-diversity (Van Diepen et al., 2011; Xiong et al., 2012; Zhu et al., 2016). Furthermore, some researchers have suggested that the change of community structure (β-diversity) was typically correlated with shifts in functional behavior (Van Diepen et al., 2011; Fierer et al., 2013; Griffiths and Philippot, 2013). We speculated that the function responds sensitively and rapidly in the environment, which shows through β-diversity rather than α-diversity. In summary, these results suggest that the microbial community functions and structure respond initially to the environment variations, and then it takes a longer time for microbial α-diversity to change in the saline-alkali soils.

It has been a great challenge to dissect the association between microbial community structure and soil ecosystem. In this study, we used co-occurrence network analysis based on correlation to thoroughly dissect the microbial associations under salinity (Barberán et al., 2012; Faust and Raes, 2012; Friedman and Alm, 2012). Network analyses were conducted to reveal positive and negative interactions among different OTUs. A microbial community in the low-salinity soil was found to have a network with higher connectivity, suggesting more operational community with a greater number of functionally interrelated members (10 major modules) compared to the microbial community network in the high-salinity soil (7 major modules) (Figure 5). Given that the highly connected microbes within a module that co-occur might share similar ecological characteristics within communities (Chaffron et al., 2010; Williams et al., 2014; Yao et al., 2014), our results suggest that the high-salinity soil could harbor less ecologically similar functional groups.

Another important benefit of network analysis in microbial ecology studies is the ability to identify central organisms in maintaining soil ecosystems, according to the network theory, the “hub” species, as hotspots of connections in the microbial network, are the most important mediators for the complicated interactions among different species constituting the soil ecosystem (Montoya et al., 2006). According to the method described by the previous study (Zhu et al., 2018), 10 nodes with the most edges were defined as network hubs. *Planctomycetes*, *Proteobacteria*, and *Bacteroidetes* were the predominant phyla occupying 63.3% of the network hubs among the different salinity treatments (Figure 6). Ma and Gong (2013) reported that six phyla (*Proteobacteria*, *Actinobacteria*, *Firmicutes*, *Acidobacteria*, and *Bacteroidetes*) contained 90% of the bacterial sequences in saline soils; here, we detected five out of the six major phyla, with the exception for *Acidobacteria*. *Proteobacteria*, one common bacterial taxa in saline soil identified by a previous study (Valenzuela-Encinas et al., 2009), was the common denominator in our experimental sites, being especially dominant in highly saline soils, with 50% frequency in the indicators. In addition, *Proteobacteria* was reported as “salinity related” in a previous study (Yang et al., 2018), our results confirmed this finding, as *Rhodospirillaceae* and *Marinicella*, assigned to *Proteobacteria*, had good correlation with salt concentration. In addition, *Firmicutes* can also be considered special indicators specifically for the high salinity rate soil, which was absent in various hyper saline environments in previous studies (Demergasso et al., 2004; Ramette, 2007). *Bacillus* stands out among the prevailing genera assigned to *Firmicutes*, as it has shown to be an important resource for exploring halophilic enzymes and metabolic pathways for pollutant remediation in saline soil (Liszka et al., 2012). Species within *Proteobacteria* and *Firmicutes* may be good indicators, reflecting changes of the main microbial groups in saline-alkali soil. Although other studies have reported *Gemmatimonadetes* and *Bacteroidetes* to be an important participant in biogeochemical transformations in soils under salinity (Fierer et al., 2012; Ma and Gong, 2013), they were not detected in the high salinity soils of the current study. Different regions form different ecological environments, resulting in various microbial compositions. Therefore, our result suggests the requirement of future study on a wide range of spatial scales.

Our examination on the variability of the microbial community in saline soils successfully revealed microbial community subdivision across micro-environmental gradients. We expect future studies using metagenome sequencing data could identify similar patterns of bacterial composition variation at finer taxonomic resolution.

## Conclusion

Taken together, this study presents an attempt to explore bacterial composition in saline-alkali soils across Jilin and Heilongjiang provinces. We show that bacterial β-diversity and community structure correlate with the salt gradient. We demonstrate that EC, instead of pH predicts bacterial community structure in saline-alkaline soils. Microbes belonging to the phyla *Proteobacteria* and *Firmicutes* were predominant and may be good groups of indicator species, reflecting changes of the main microbial groups in saline-alkali soil. Our results revealed local geochemical features as driving force of bacterial composition in the soil, whereas the EC as a key dominant factor in regulating microbial composition at a regional spatial scale. Correlating population of microbes with environmental parameters could facilitate reconstructing the formation of bacterial communities under specific environmental conditions like salinity. In this study, we provide a framework for future research to deeply analyze microbial composition in extreme environments.

## Data Availability Statement

The datasets generated for this study can be found in the NCBI Sequence Read Archive (SRA) database/SRP172399.

## Author Contributions

DW and FC contributed to the conception of the study. SW and LS contributed significantly to analysis and manuscript preparation. WL, XH, WZ, and JB performed the collection of soil samples and data analyses. NL revised the manuscript. LC played an important role in interpreting the results. CZ helped perform the analysis with constructive discussions.

### Conflict of Interest

The authors declare that the research was conducted in the absence of any commercial or financial relationships that could be construed as a potential conflict of interest.
