# Supplementary material for: Exploring Soil Factors Determining Composition and Structure of the Bacterial Communities in Saline-Alkali Soils of Songnen Plain
Source: Front Microbiol. 2020 Jan 14;10:2902. doi: 10.3389/fmicb.2019.02902 (PMC6972583; doi:10.3389/fmicb.2019.02902)
Supplement: Supplementary file 1 [file Table_1.DOCX]

Supplementary Material

Supplementary Table 1. Physical and chemical properties of collected soils.

| Sample | Latitude | Longitude | pH | Cl^-^(g/kg) | SO_4_^2-^(g/kg) | HCO_3_^-(^g/kg) | CO_3_^2-^(g/kg) | Ca^2+^(g/kg) | Mg^2+^(g/kg) | Na^+^(g/kg) | Exchangeable Na^+^(cmol/kg) | SOC(mg/g) | EC(ms/cm) | SAR | Main vegatation types |
| --- | --- | --- | --- | --- | --- | --- | --- | --- | --- | --- | --- | --- | --- | --- | --- |
| Jc1 | 44.249 | 123.857 | 10.26 | 0.26 | 0.83 | 0.99 | 0.01 | 0.24 | 0.27 | 0.12 | 19.30 | 2.26 | 0.41 | 0.16 | *Leymus chinensis* |
| Js1 | 45.056 | 124.634 | 9.93 | 0.27 | 0.96 | 2.38 | 0.02 | 0.64 | 0.20 | 0.23 | 32.30 | 4.58 | 0.71 | 0.25 | *Phragmites australis* |
| Jz2 | 45.959 | 123.705 | 10.37 | 0.27 | 0.70 | 2.76 | 0.02 | 0.24 | 0.39 | 0.38 | 32.00 | 2.54 | 0.75 | 0.47 | *Chloris virgata*, *Leymus chinensis* |
| Hda2 | 46.219 | 124.793 | 10.14 | 0.64 | 0.96 | 3.39 | 0.20 | 1.20 | 0.39 | 0.45 | 35.10 | 4.80 | 0.85 | 0.35 | *Chloris virgata, Suaeda glauca* |
| Hz1 | 46.029 | 125.835 | 10.20 | 2.22 | 3.17 | 3.66 | 0.21 | 1.20 | 0.32 | 0.55 | 42.90 | 5.00 | 1.34 | 0.44 | *Chloris virgata* |
| Jz1 | 45.807 | 123.823 | 10.61 | 0.36 | 0.60 | 2.98 | 0.03 | 0.56 | 0.15 | 0.51 | 43.00 | 2.65 | 0.86 | 0.61 | *Leymus chinensis* |
| Jd1 | 45.594 | 123.949 | 10.51 | 0.80 | 0.67 | 2.76 | 0.03 | 0.40 | 0.15 | 0.88 | 56.00 | 3.02 | 2.02 | 1.19 | *Chloris virgata*, *Leymus chinensis* |
| Jt1 | 44.635 | 123.139 | 10.66 | 0.49 | 1.20 | 2.56 | 0.03 | 0.55 | 0.35 | 0.51 | 46.10 | 6.50 | 1.76 | 0.54 | *Leymus chinensis,Chloris virgata* |
| Hz3 | 45.751 | 125.365 | 10.27 | 2.13 | 4.80 | 6.89 | 2.52 | 0.96 | 1.37 | 0.67 | 47.30 | 2.80 | 1.79 | 0.44 | *Aster subulatus* |
| Hdu1 | 46.32 | 124.137 | 10.26 | 0.89 | 12.48 | 1.21 | 0.73 | 0.20 | 0.07 | 0.45 | 40.30 | 2.77 | 1.30 | 0.86 | *Chloris virgata,Tripolium vulgare* |
| Hdu2 | 46.546 | 124.283 | 10.27 | 0.55 | 18.05 | 6.61 | 1.54 | 0.21 | 0.41 | 0.74 | 38.40 | 3.24 | 1.83 | 0.94 | *Carex ischnostachya* |
| Jc3 | 44.314 | 123.423 | 10.45 | 0.81 | 0.91 | 2.26 | 0.02 | 0.17 | 0.44 | 0.55 | 36.90 | 3.21 | 1.90 | 0.70 | *Chloris virgata* |
| Jt2 | 44.778 | 123.211 | 9.92 | 0.64 | 1.06 | 0.73 | 0.01 | 0.16 | 0.05 | 0.94 | 33.80 | 2.46 | 2.99 | 2.06 | *Chloris virgata* |
| Jq2 | 44.876 | 123.703 | 10.59 | 0.89 | 1.63 | 1.73 | 0.03 | 0.32 | 0.06 | 1.48 | 56.70 | 4.62 | 3.24 | 2.40 | *Chloris virgata* |
| Jc2 | 44.244 | 123.819 | 10.64 | 0.44 | 1.08 | 0.50 | 0.04 | 0.24 | 0.02 | 0.68 | 39.70 | 2.32 | 2.63 | 1.32 | *Chloris virgata* |
| Js2 | 45.021 | 124.586 | 10.35 | 0.39 | 1.10 | 1.81 | 0.03 | 0.44 | 0.12 | 0.59 | 45.30 | 1.93 | 2.05 | 0.79 | *Salsola soda Linn., Leymus chinensis* |
| Hda3 | 46.128 | 124.846 | 10.16 | 0.71 | 21.60 | 6.22 | 1.02 | 0.34 | 0.58 | 0.67 | 37.70 | 2.04 | 2.22 | 0.70 | *Leymus chinensis* |
| Jd2 | 45.579 | 123.942 | 10.51 | 0.50 | 0.79 | 1.65 | 0.03 | 0.38 | 0.06 | 0.64 | 54.30 | 5.34 | 2.06 | 0.96 | *Chloris virgata* |
| Js3 | 44.939 | 124.598 | 10.60 | 0.44 | 1.10 | 2.79 | 0.04 | 0.72 | 0.15 | 0.59 | 45.80 | 1.70 | 1.90 | 0.64 | *Suaeda glauca* |
| Hl2 | 47.169 | 124.668 | 10.30 | 0.44 | 14.21 | 7.17 | 1.44 | 0.72 | 0.29 | 0.72 | 59.40 | 7.18 | 2.03 | 0.72 | *Leymus chinensis,Chloris virgata* |
| Ha1 | 46.16 | 125.053 | 10.29 | 0.89 | 0.48 | 4.45 | 1.98 | 0.40 | 0.27 | 1.22 | 42.40 | 3.06 | 2.88 | 1.50 | *Chloris virgata,Leymus chinensis,Setaria glauca* |
| Hz5 | 45.634 | 124.972 | 9.89 | 3.20 | 4.70 | 0.88 | 0.34 | 0.36 | 0.05 | 0.85 | 42.70 | 4.59 | 2.99 | 1.33 | *Chloris virgata* |
| Jq1 | 44.868 | 123.641 | 9.97 | 1.01 | 1.23 | 1.16 | 0.01 | 0.23 | 0.10 | 1.27 | 47.50 | 7.58 | 4.24 | 2.20 | *Chloris virgata* |
| Hz6 | 45.727 | 124.782 | 10.40 | 1.78 | 13.44 | 5.25 | 2.70 | 2.40 | 0.73 | 1.43 | 65.80 | 6.59 | 4.43 | 0.81 | *Chloris virgata* |
| Jt3 | 44.772 | 123.333 | 10.42 | 0.43 | 1.19 | 2.04 | 0.04 | 0.08 | 0.05 | 1.39 | 54.00 | 1.53 | 6.58 | 3.87 | *Chloris virgata* |
| Hl1 | 46.989 | 124.591 | 10.01 | 2.49 | 1.73 | 4.90 | 3.96 | 0.40 | 0.10 | 2.01 | 51.70 | 4.56 | 5.40 | 2.85 | *Imperata cylindrica, Phragmites australis, Leymus chinensis* |
| Ha2 | 46.169 | 125.073 | 8.84 | 7.17 | 1.00 | 1.83 | 0.00 | 0.50 | 0.29 | 2.57 | 39.80 | 15.43 | 8.41 | 2.89 | *Chloris virgata, Suaeda glauca* |
| Hda1 | 46.434 | 124.821 | 10.13 | 4.47 | 3.84 | 1.46 | 2.28 | 0.22 | 0.21 | 2.11 | 36.90 | 3.99 | 7.31 | 3.23 | *Suaeda glauca* |
| Hda4 | 46.123 | 124.87 | 10.12 | 10.47 | 4.07 | 2.32 | 2.82 | 0.31 | 0.02 | 2.45 | 48.90 | 4.77 | 10.87 | 4.29 | *Chloris virgata, Suaeda glauca* |

SOC: the organic carbon

EC: electrical conductivity

The Na^+^ absorption ratio (SAR) was calculated as: SAR = [Na^+^]/ (0.5[Ca^2+^ + Mg^2+^]1/2)

Supplementary Table 2. Classifications of indicator OTUs in network analysis.

|  |  | Phlya | Class | Order | Family | Genus |
| --- | --- | --- | --- | --- | --- | --- |
| L treatment | OTU106 | *Planctomycetes* | *Planctomycetacia* | *Planctomycetales* | *Planctomycetaceae* | Unclassified |
|  | OTU63 | *Planctomycetes* | *Planctomycetacia* | *Planctomycetales* | *Planctomycetaceae* | Unclassified |
|  | OTU87 | Unclassified | Unclassified | Unclassified | Unclassified | Unclassified |
|  | OTU513 | *Planctomycetes* | *Planctomycetacia* | *Planctomycetales* | *Planctomycetaceae* | Unclassified |
|  | OTU673 | *Proteobacteria* | Unclassified | Unclassified | Unclassified | Unclassified |
|  | OTU1147 | *Bacteroidetes* | *Bacteroidia* | *Bacteroidales* | *Bacteroidaceae* | *Bacteroides* |
|  | OTU941 | *Gemmatimonadetes* | *Gemmatimonadetes* | Unclassified | Unclassified | Unclassified |
|  | OTU430 | *Chloroflexi* | Unclassified | Unclassified | Unclassified | Unclassified |
|  | OTU781 | *Bacteroidetes* | *Cytophagia* | *Cytophagales* | *Cytophagaceae* | *Hymenobacter* |
|  | OTU98 | *Proteobacteria* | *Gammaproteobacteria* | *Pseudomonadales* | *Pseudomonadaceae* | *Pseudomonas* |
| M treatment | OTU777 | *Proteobacteria* | Unclassified | Unclassified | Unclassified | Unclassified |
|  | OTU311 | *Gemmatimonadetes* | *Gemmatimonadetes* | Unclassified | Unclassified | Unclassified |
|  | OTU70 | *Synergistetes* | *Synergistia* | *Synergistales* | *Synergistaceae* | Unclassified |
|  | OTU118 | *Planctomycetes* | *Planctomycetacia* | *Planctomycetales* | *Planctomycetaceae* | Unclassified |
|  | OTU511 | Unclassified | Unclassified | Unclassified | Unclassified | Unclassified |
|  | OTU945 | *Bacteroidetes* | Unclassified | Unclassified | Unclassified | Unclassified |
|  | OTU19 | *Planctomycetes* | *Phycisphaerae* | Unclassified | Unclassified | Unclassified |
|  | OTU508 | *Bacteroidetes* | *Flavobacteria* | *Flavobacteriales* | *Flavobacteriaceae* | *Gillisia* |
|  | OTU10 | *Bacteroidetes* | *Cytophagia* | Unclassified | Unclassified | Unclassified |
|  | OTU114 | *Acidobacteria* | *Holophagae* | Unclassified | Unclassified | Unclassified |
| H treatment | OTU144 | *Bacteroidetes* | *Flavobacteria* | *Flavobacteriales* | *Flavobacteriaceae* | *Chryseobacterium* |
|  | OTU555 | *Proteobacteria* | *Gammaproteobacteria* | *Legionellales* | *Coxiellaceae* | *Aquicella* |
|  | OTU274 | *Proteobacteria* | *Betaproteobacteria* | *Nitrosomonadales* | *Nitrosomonadaceae* | *Nitrosomonas* |
|  | OTU713 | *Proteobacteria* | *Alphaproteobacteria* | Unclassified | Unclassified | Unclassified |
|  | OTU467 | *Firmicutes* | *Erysipelotrichia* | *Erysipelotrichales* | *Erysipelotrichaceae* | *Unclassified* |
|  | OTU736 | *Firmicutes* | *Bacilli* | *Bacillales* | Unclassified | Unclassified |
|  | OTU161 | *Actinobacteria* | *Nitriliruptoria* | *Nitriliruptorales* | *Nitriliruptoraceae* | *Nitriliruptor* |
|  | OTU491 | *Chloroflexi* | *Anaerolineae* | *Anaerolineales* | *Anaerolineaceae* | Unclassified |
|  | OTU572 | *Proteobacteria* | *Betaproteobacteria* | *Nitrosomonadales* | *Nitrosomonadaceae* | *Nitrosomonas* |
|  | OTU783 | *Proteobacteria* | *Gammaproteobacteria* | *Oceanospirillales* | *Halomonadaceae* | *Halomonas* |

**Supplementary Table 3.** Monte Carlo permutation test for CCA of community structure

|  | CCA1 | CCA2 | r^2^ | P |
| --- | --- | --- | --- | --- |
| CO_3_^2-^ | 0.978 | 0.21 | 0.619 | 0.001*** |
| SO_4_^2-^ | -0.54 | -0.84 | 0.335 | 0.043* |
| pH | 0.923 | 0.385 | 0.046 | 0.425 |
| Mg^2+^ | 0.804 | 0.594 | 0.11 | 0.174 |
| Exchangeable Na^+^ | 0.81 | -0.59 | 0.161 | 0.086 |
| SOC | 0.237 | 0.971 | 0.07 | 0.272 |
| Ca^2+^ | 0.358 | 0.934 | 0.048 | 0.39 |
| SAR | -0.35 | -0.94 | 0.6 | 0.001*** |
| HCO_3_^-^ | 0.318 | 0.948 | 0.369 | 0.002** |
| Cl^-^ | 0.617 | -0.79 | 0.179 | 0.055 |
| Na^+^ | -0.99 | -0.12 | 0.557 | 0.011* |
| EC | -0.28 | -0.96 | 0.672 | 0.001*** |

SOC: the organic carbon

EC: electrical conductivity

The Na^+^ absorption ratio (SAR) was calculated as: SAR = [Na^+^]/ (0.5[Ca^2+^ + Mg^2+^]1/2)

* The correlations are significant at *P* < 0.05.

***The correlations are significant at *P* < 0.001.


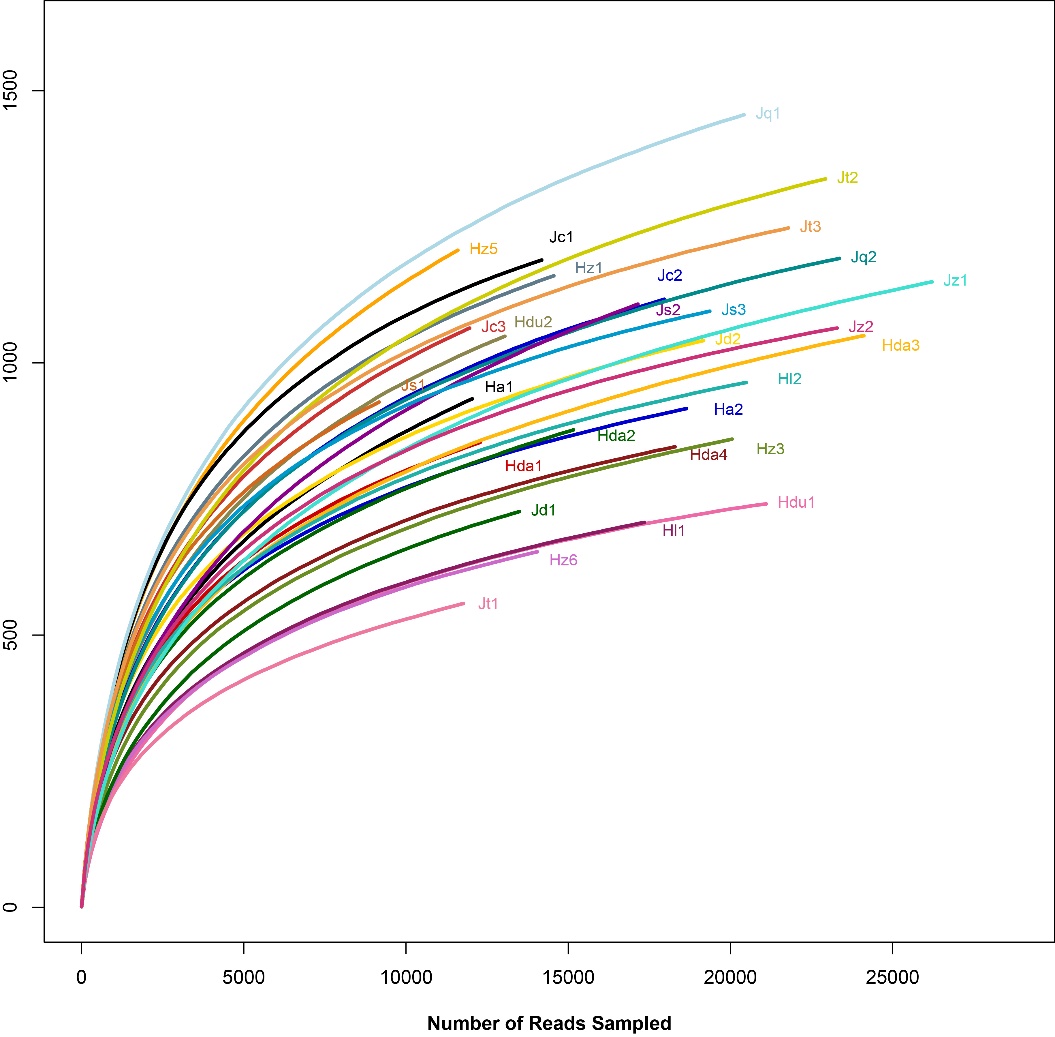


**Supplementary Figure 1.** The rarefaction curves of bacteria for different samples.


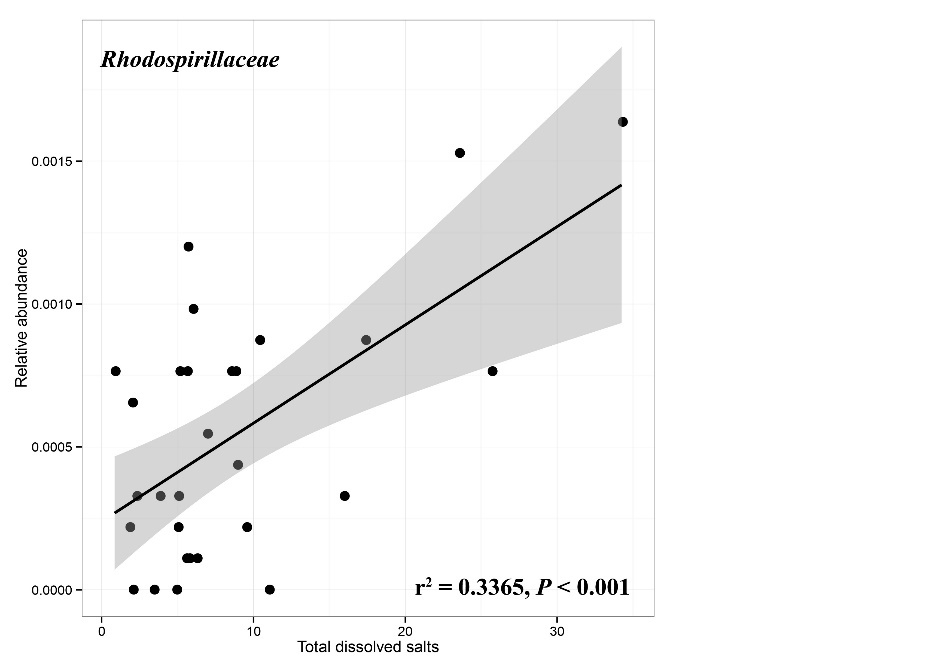

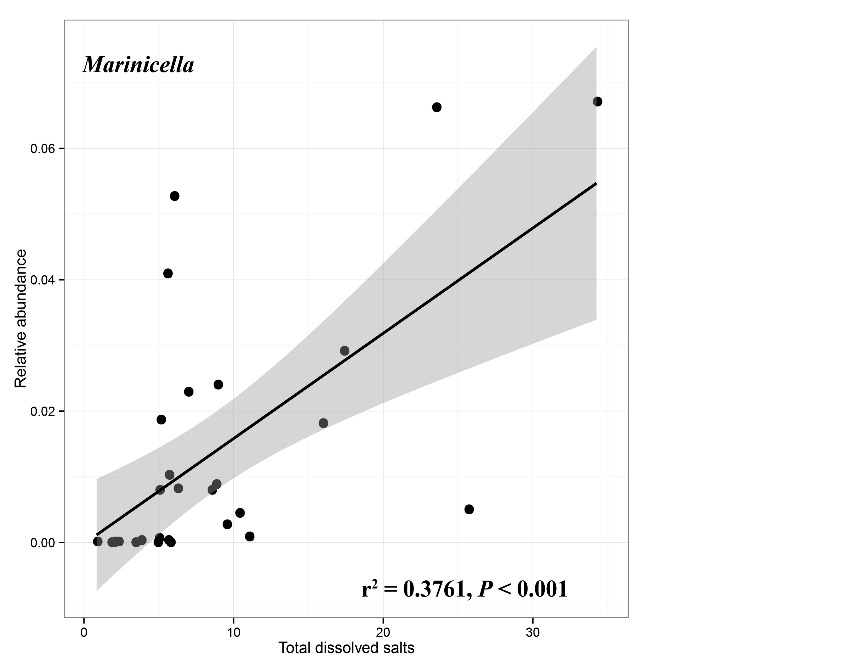


（a）

（b）

**Supplementary Figure 2.** Linear correlations between the relative abundance of different bacterial classification level and total dissolved salts. (a) At the Family level and (b) at the Genus level.
